# Efficacy and Safety of OROSOL Spray for Oral Mucositis in Children: A Randomized, Double-Blind, Placebo-Controlled Trial

**DOI:** 10.3390/biomedicines13071677

**Published:** 2025-07-09

**Authors:** Fatima-Zahra El Barche, Manon D’Almeida, Séverine Dameron, Rémi Shrivastava

**Affiliations:** Vitrobio Research Institute, ZAC de Lavaur, 63500 Issoire, France; m.d_almeida@vitrobio.com (M.D.); s.dameron@vitrobio.com (S.D.);

**Keywords:** cancer therapy, medical device, oral mucositis, oral spray, OROSOL

## Abstract

**Background**: Oral mucositis (OM) is a common and debilitating complication of cancer therapy, particularly in patients undergoing chemotherapy and radiotherapy. It significantly impairs quality of life and may necessitate the interruption of cancer treatment. This study aimed to evaluate the efficacy and safety of OROSOL, an oral spray device, in managing oral mucositis in pediatric patients undergoing chemotherapy or radiotherapy. **Methods**: This randomized, double-blind, placebo-controlled clinical trial compared OROSOL to a placebo in children with oral mucositis aged 3 to 17 years. Participants were followed for 28 days with regular medical visits. The primary endpoints were changes in the Oral Assessment Guide (OAG) scores and key symptoms (mucositis score, difficulty in oral feeding, ulceration and erythema, and pain sensation). Safety was assessed via adverse events and local tolerability. **Results**: Both groups were demographically balanced at baseline (*p* > 0.6). OROSOL demonstrated significantly greater improvements in the mucositis score beginning on Day 7 (*p* = 0.0122) and maintained superiority through Day 28 (*p* = 0.0007). Notable reductions in mucositis severity were observed, with significantly faster relief in the OROSOL group compared to the placebo (*p* < 0.001 for most timepoints). Oral feeding difficulty also showed a marked decline, with significant improvements starting from Day 5 (*p* = 0.0153). Ulceration and erythema scores significantly decreased from Day 14 onwards (*p* = 0.0188). Pain sensation showed a marked reduction from Day 14 (*p* = 0.0014). No serious adverse events were reported, and tolerability was consistent across all participants. **Conclusions**: OROSOL has a significant impact on reducing mucositis severity, oral feeding difficulty, ulceration, erythema, and pain. Coupled with its excellent safety profile, it is a valuable therapeutic option. This treatment is particularly beneficial for pediatric patients, ensuring improved comfort and recovery without notable adverse effects.

## 1. Introduction

Oral mucositis (OM) is an inflammatory lesion of the mucosa that arises as a consequence of chemotherapy or radiotherapy. This condition can significantly impact a patient’s quality of life and may necessitate discontinuation of treatment due to compromised immunity [1]. Mucosal lesions can be unpredictable, painful, and costly for both patients and caregivers [2]. This complication affects up to 40% of cancer patients undergoing chemotherapy and up to 80% of patients with head and neck cancers receiving radiotherapy [3,4,5]. Management strategies are primarily supportive and focus on alleviating symptoms, reducing the duration of lesions, and preventing secondary infections [6]. Effective pain control is a cornerstone in the treatment of OM. Topical anesthetics, such as viscous lidocaine and benzocaine gels, provide temporary relief by numbing the mucosal surfaces [7]. In more severe cases, systemic analgesics, including opioids, may be required [8]. Maintaining optimal oral hygiene reduces the risk of secondary infections and promotes healing. Saline or sodium bicarbonate rinses are widely recommended due to their non-irritating properties [9]. The use of a soft-bristled toothbrush, regular brushing, and avoidance of alcohol-based mouthwashes are standard components of supportive care protocols [10]. Secondary infections are a frequent complication of OM. Antifungal agents, such as nystatin, are commonly used in the presence of oral candidiasis. Antibacterial rinses like chlorhexidine gluconate have been evaluated, but their routine use remains controversial due to potential mucosal irritation [11]. Oral cryotherapy, which involves the use of ice chips during chemotherapy administration (especially with agents like 5-fluorouracil or melphalan), reduces mucosal blood flow and drug exposure, thereby decreasing OM severity [12]. Low-level laser therapy (LLLT) has gained traction due to its anti-inflammatory and analgesic effects. Evidence from randomized controlled trials supports its use in both the prevention and treatment of OM in patients receiving radiotherapy for head and neck cancer [13,14]. Mucoadhesive products and bioadherent gels form a protective film over mucosal lesions, thereby reducing pain and facilitating oral intake. Some of these agents also contain ingredients that promote mucosal healing [15].

Despite these advances, the management of OM remains a clinical challenge, particularly in pediatric populations, where tolerability, safety, and ease of administration are paramount. There is a pressing need for well-tolerated, effective, and non-systemic therapies that can provide both symptomatic relief and mucosal protection.

The present study investigates the efficacy and safety of OROSOL, an internationally patented oral spray designed with a glycerol-based formulation incorporating plant-derived polymers, designed to form a protective and osmotic film [16]. Glycerol acts as a hyperosmotic and film-forming agent. This formulation exhibits a high osmotic capacity (up to 10 times greater than that of seawater) while simultaneously forming a stable, long-lasting film on the oral mucosa, with adhesion lasting for several hours. Upon application to ulcerated or inflamed areas, OROSOL forms a flexible, resistant barrier that exerts a dual mechanism of action: (1) an osmotic effect that draws hypotonic fluid from the submucosal tissue and (2) a barrier effect that isolates the injured mucosa from external irritants. The osmotic action facilitates the mechanical detachment and removal of surface contaminants, including bacteria, necrotic cells, infiltrated chemicals, and free protein molecules, without the use of chemical agents. This cleansing process promotes the creation of a favorable microenvironment for cell regeneration and tissue repair. Acting locally without systemic absorption, OROSOL provides effective protection against secondary aggressors and contributes to symptomatic relief of oral mucositis, including pain, inflammation, and impaired oral function. Its purely mechanical mode of action makes it a well-tolerated therapeutic option, particularly suitable for vulnerable populations such as pediatric patients. To evaluate the safety and clinical efficacy of OROSOL in the management of oral mucositis, a randomized, double-blind, placebo-controlled trial was conducted. This study enrolled pediatric patients aged 3 to 17 years, providing a robust framework for assessing the therapeutic benefits of the device in a vulnerable population frequently affected by mucosal complications during cancer therapy.

## 2. Methods

### 2.1. Study Design and Ethics

This study was designed as a comparative, randomized, double-blind, parallel-group, interventional clinical trial conducted in accordance with the principles of the International Council for Harmonisation (ICH) Good Clinical Practice (GCP) guidelines, Indian GCP, and ISO 14155 guidelines. The study was conducted at a single clinical site: Ratnaraj Clinic and Nursing Home, Vitthal Nagar, Karjat, District Raigad, Maharashtra, India. The clinical study was registered under the registration number CTRI/2024/07/072435 on 6 September 2024. The protocol was approved by the appropriate independent ethics committee, and all participants or their legal guardians provided written informed consent prior to enrollment. The trial was sponsored by Polytrap Pharma Pvt. Ltd. (Indore, India) and operationally managed by Mudra Clincare, Navi Mumbai, India.

### 2.2. Study Population

The initial study population included 29 children aged 3 to 17 years. Following data verification, only 21 patients had complete datasets for the entire study duration. Among the excluded patients, five were categorized as screen failure and three were excluded by the general practitioner (GP). Consequently, the final dataset comprised 21 patients.

Participants were divided into two groups, one received OROSOL (*n* = 11) and the other received a placebo (*n* = 10), resulting in a nearly balanced distribution between the groups despite the exclusions.

### 2.3. Interventions and Randomization

Participants were randomized in a 1:1 ratio to receive either the investigational product (OROSOL) or a placebo comparator spray. Randomization was performed using SAS Version 9.1.3 with block randomization, ensuring balanced allocation across the age range (3–17 years) and blinding of all investigators, patients, and caregivers to treatment allocation.

### 2.4. Investigational Device

The investigational product, OROSOL, is an oral spray supplied in a 20 mL aluminum container. Its formulation includes glycerol, honey, water (aqua), and Mucocyanidin (derived from extracts of Vaccinium macrocarpon and Vaccinium myrtillus).

The placebo comparator was a thickened water-based solution designed to match the viscosity and appearance of the OROSOL spray. It was identical in packaging and mode of administration and contained solagum, potassium sorbate, aqua, sodium benzoate, and citric acid. Both formulations were produced and supplied by Vitrobio (Issoire, France). Both OROSOL and placebo sprays were administered as 4 to 5 topical applications 3 to 4 times a day.

### 2.5. Study Procedures and Assessments

Following the initial screening to confirm eligibility, baseline data were collected, including demographic characteristics, scores from the Oral Assessment Guide (OAG), and initial symptom evaluations. The OAG is an assessment scale used by clinicians to evaluate changes in the oral cavity. It encompasses eight categories relevant to oral mucositis, each scored on a scale from 1 to 3, where a score of “1” represents normal function and “3” indicates severe impairment or tissue breakdown.

Participants were evaluated at multiple predefined time points: 24 h after the first dose (Day 1) and subsequently on Days 3, 5, 7, 14, 21, and 28. At each visit, symptom assessments were conducted, with scores recorded for mucositis severity, oral feeding difficulty, ulceration and erythema, and pain sensation. For younger participants, these assessments were completed by parents or caregivers.

Treatment adherence was monitored using structured patient diaries, which were provided at enrollment and filled out daily by the participants’ parents or legal guardians. The diaries documented the number and timing of OROSOL or placebo spray applications, any missed doses, and observations related to ease of administration or acceptance by the child. Investigators reviewed the diaries at each follow-up visit, and any deviations from the protocol were recorded. This adherence monitoring was used to support the interpretation of efficacy data and ensure compliance with the prescribed regimen.

Throughout the study period, all adverse events (AEs) and serious adverse events (SAEs) were continuously monitored and documented. The use of concomitant medications was generally prohibited unless antibiotic treatment was considered clinically necessary by the attending physician. Participants who used prohibited medications or failed to adhere to the study protocol were withdrawn from the trial.

### 2.6. Blinding and Unblinding

Both OROSOL and placebo products were identical in appearance, packaging, and labeling, with unique product codes known only to the sponsor’s designated personnel. Patients, investigators, and clinical staff remained blinded to the treatment allocation until database lock. Emergency unblinding was permissible if necessary for patient safety, and any unblinding event was documented and justified. To preserve blinding integrity, the placebo was designed to closely mimic the organoleptic properties of OROSOL, including texture and spray pattern. Although the formulations differed slightly in taste, the parallel-group design ensured that participants were only exposed to one formulation and therefore had no basis for comparison.

### 2.7. Statistical Analysis

The investigated dataset included patients who completed all visits and assessments. Baseline demographic and clinical characteristics were compared between treatment groups using appropriate statistical tests (Student’s *t*-test, Wilcoxon rank-sum test, and Fisher’s exact test) to confirm population homogeneity. Efficacy endpoints were analyzed using two-way ANOVA for repeated measures, followed by post hoc tests (*t*-test or Wilcoxon) for pairwise comparisons at each time point. Adverse event frequency was analyzed using chi-square or Fisher’s exact test.

## 3. Results

### 3.1. Demographic Outcomes

A total of 21 patients were included in the efficacy and safety analysis, with 11 patients receiving OROSOL and 10 patients receiving the placebo comparator. Baseline demographic characteristics, including age, sex distribution, height, and weight of the overall population were well-balanced between the two groups, as summarized in Table 1.

The comprehensive comparison of demographic characteristics between Group R (placebo) and Group T (OROSOL) revealed no statistically significant differences. The Chi2 Test applied to sex distribution yielded a non-significant *p*-value, indicating no variation in the proportion of males and females between the two groups. Similarly, independent Student’s *t*-tests conducted for age, height, and weight showed non-significant *p*-values, confirming the absence of significant differences in the mean values of these variables between groups. These results collectively demonstrate demographic homogeneity between the two groups, ensuring comparability across key baseline characteristics. In addition to demographic comparability, the two study groups were also balanced in terms of baseline clinical severity of mucositis. All patients met the inclusion criteria, which required either treatment with chemotherapy for hematological cancers (such as leukemia or lymphoma) or treatment with radiotherapy or radio-chemotherapy (e.g., cisplatin or cetuximab) for head and neck cancers. In both cases, eligible patients were required to present with mucositis graded 2 to 4 according to the WHO/NCI-CTCAE criteria. At baseline, the distribution of mucositis severity was comparable between groups. In the OROSOL treatment group (Group T), 58.3% had Grade 2 mucositis, while 41.7% had Grade 3 mucositis. In the placebo group (Group R), 50% presented with Grade 2 mucositis and 50% with Grade 3. This distribution demonstrates a well-balanced allocation of baseline clinical severity between the two arms, supporting the internal validity of the randomization and reducing the likelihood that differences in initial mucositis grade influenced treatment outcomes.

### 3.2. Primary Endpoint

#### Symptom Scores

a Mucositis Severity Score

**Figure 1:**
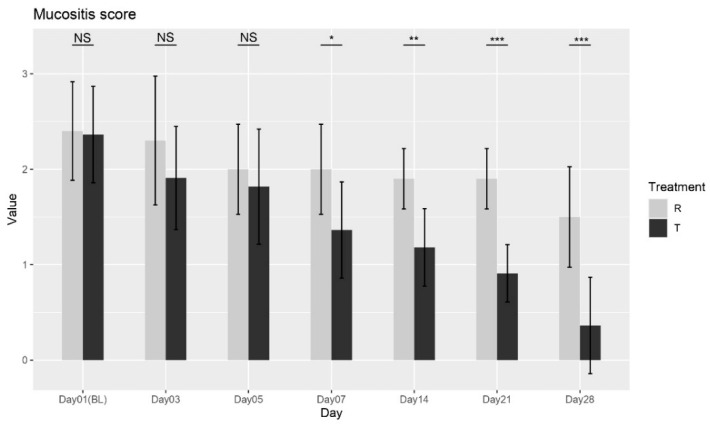
Mucositis severity score variation over time for Treatment T and Treatment R. NS = non-significant, * = *p* < 0.01, ** = *p* < 0.001, *** = *p* < 0.0001.

The Wilcoxon rank-sum test was performed between the two treatments at each visit, and no significant differences were found at Baseline (Day 01), Day 03, or Day 05. However, a significant difference was observed at Day 07 (*p* = 0.0122), and this trend continued with significant differences at Day 14 (*p* = 0.0015), Day 21 (*p* < 0.0001), and Day 28 (*p* = 0.0007), as shown in Figure 1. These results confirm that Treatment T (OROSOL) led to significant improvements in mucositis severity compared to Treatment R (Placebo), with benefits becoming apparent from Day 07 and continuing through Day 28.

The ANOVA analysis further corroborated these findings, showing that both treatment and day had significant effects on mucositis severity, with the interaction between these factors indicating that the treatment’s effect varies depending on the time point. Collectively, the Wilcoxon test and ANOVA analysis provide strong evidence that Treatment T is more effective than Treatment R in reducing mucositis severity over time, supporting its efficacy in managing mucositis.

b Oral Feeding Difficulty

**Figure 2:**
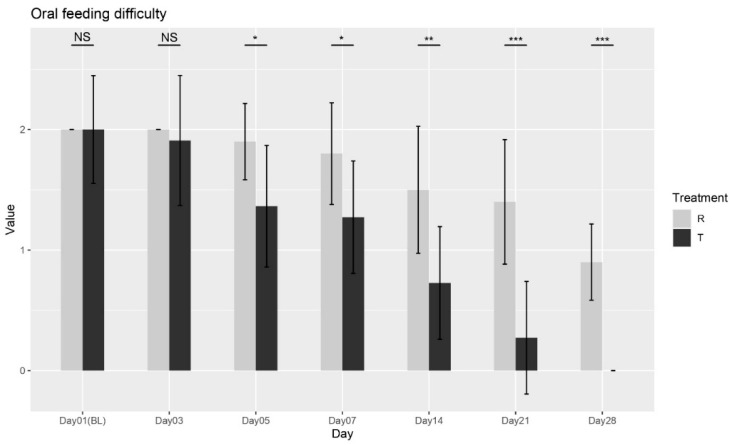
Oral feeding difficulty scores over time for Treatment T and Treatment R. NS = non-significant, * = *p* < 0.01, ** = *p* < 0.001, *** = *p* < 0.0001.

The Wilcoxon rank-sum test was conducted between the two treatments at each visit. No significant differences were observed at Baseline (Day 01) or Day 03 (*p* = 1; *p* = 0.6025). However, significant differences were found starting from Day 05 (*p* = 0.0153), with continued improvements observed at Day 07 (*p* = 0.020), Day 14 (*p* = 0.0049), Day 21 (*p* = 0.0005), and Day 28 (*p* < 0.0001), as shown in Figure 2. These results confirm that Treatment T led to significant improvements in oral feeding difficulty compared to Treatment R from Day 05 onward, with continued benefits through Day 28. Overall, the findings suggest that Treatment T is more effective in reducing feeding difficulty over time.

c Ulceration and Erythema Score

**Figure 3:**
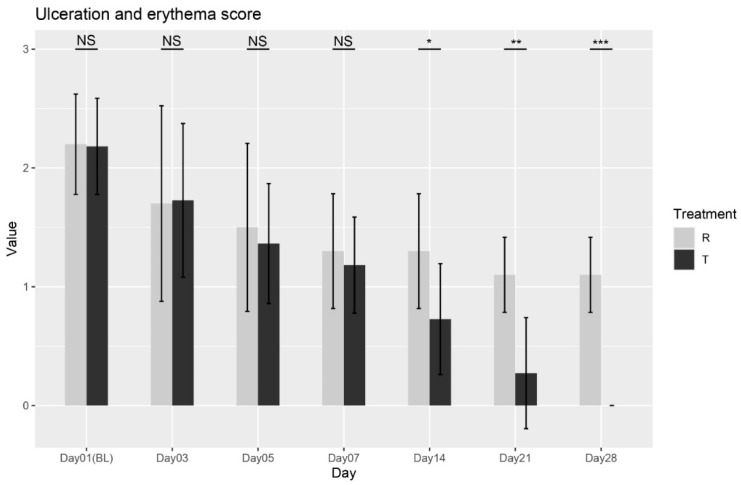
Ulceration and erythema scores variation over time for Treatment T and Treatment R. NS = non-significant, * = *p* < 0.01, ** = *p* < 0.001, *** = *p* < 0.0001.

The Wilcoxon rank-sum test was conducted between the two treatments at each visit. No significant differences were found at Baseline (Day 01), Day 03, Day5, or Day7 (*p* = 0.9588; *p* = 0.8478; *p* = 0.7726; *p* = 0.5673). However, significant differences were observed from Day 14 onward (*p* = 0.0188; *p* = 0.0009; *p* < 0.0001), confirming that Treatment T led to significant improvements in ulceration and erythema compared to Treatment R starting from Day 14 through Day 28, as shown in Figure 3. The findings indicate that Treatment T is more effective in reducing ulceration and erythema over time. Overall, these results provide strong evidence for the effectiveness of Treatment T in managing ulceration and erythema.

d Pain Sensation Score

**Figure 4:**
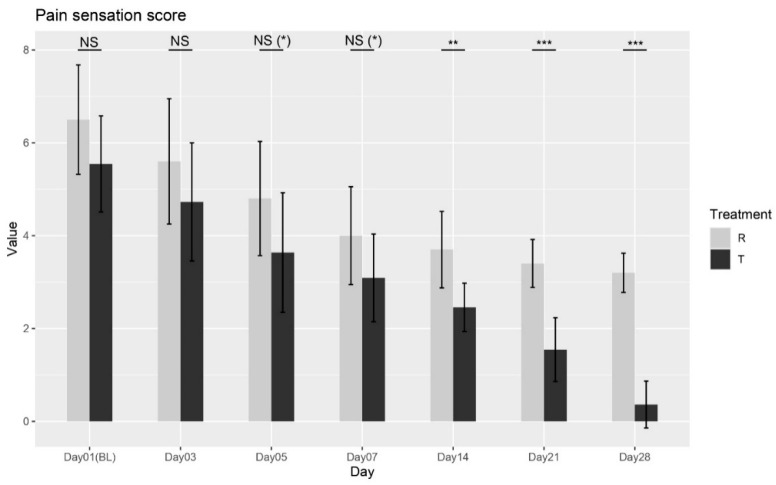
Pain sensation score variation over time for Treatment T and Treatment R. NS = non-significant, * = *p* < 0.01, ** = *p* < 0.001, *** = *p* < 0.0001.

The Wilcoxon rank-sum test was performed between the two treatments at each visit. No significant differences were observed at Baseline (Day 01), Day 03, Day 05, or Day 07 (*p* = 0.0997; *p* = 0.1802; *p* = 0.0561; *p* = 0.0589). However, significant differences were observed starting from Day 14 through Day 28 (*p* = 0.0014; *p* = 0.0001; *p* < 0.0001), as shown in Figure 4, confirming that Treatment T led to significant improvements in pain sensation compared to Treatment R. These results indicate that Treatment T is more effective in reducing pain sensation over time.

Overall, the ANOVA and Wilcoxon tests provide strong evidence supporting the effectiveness of Treatment T in managing pain sensation.

### 3.3. Secondary Endpoint

#### Oral Assessment Guide (OAG)

**Figure 5:**
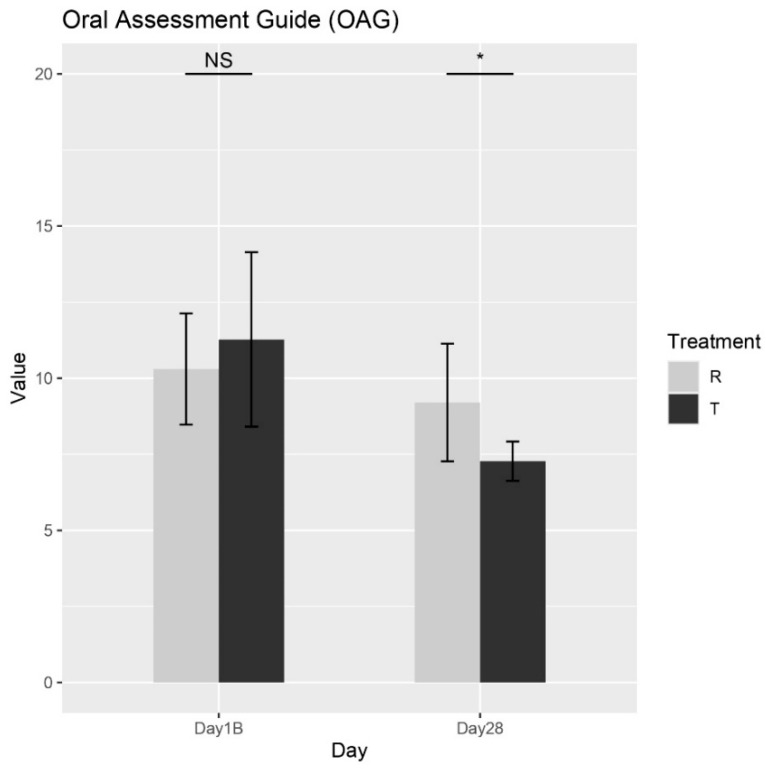
The mean total OAG scores for each treatment across different days. NS = non-significant, * = *p* < 0.01.

According to the Wilcoxon rank-sum test, a significant difference in the effects of Treatments R (placebo) and T (OROSOL) on the OAG score at Day 28 was observed, as shown in Figure 5. Specifically, Treatment T demonstrated a significantly greater reduction in symptoms compared to Treatment R at this time point. As anticipated, no significant differences were observed at baseline, which is expected since neither treatment had been administered prior to that time. These results strongly support the efficacy of Treatment T in alleviating oral mucositis symptoms in pediatric patients.

### 3.4. Safety and Tolerability

Both treatments were well tolerated, with no serious adverse events reported. There was no significant difference between groups in the incidence of local irritation or other mild adverse effects (*p* = 0.9455). No allergic reactions were observed in either group.

## 4. Discussion

Oral mucositis (OM) is a frequent and debilitating side effect of cancer therapy that significantly impairs quality of life, especially in pediatric patients undergoing chemotherapy or radiotherapy. Effective management strategies must relieve pain, accelerate mucosal healing, and be well-tolerated. In this randomized, placebo-controlled clinical trial, OROSOL demonstrated significant clinical benefits across multiple OM endpoints, reducing mucositis severity, ulceration, pain, and feeding difficulty while maintaining excellent tolerability.

Several therapies are currently considered gold standards for OM management in specific clinical contexts. Palifermin, a recombinant keratinocyte growth factor, is FDA-approved for preventing severe OM in patients undergoing high-dose chemotherapy and hematopoietic stem cell transplantation. In a pivotal trial, palifermin reduced both the incidence and duration of WHO Grade 3–4 OM compared to placebo [17]. However, its use is limited in practice due to intravenous administration, high cost, and limited pediatric data.

Low-level laser therapy (LLLT), also known as photobiomodulation, is strongly recommended by MASCC/ISOO guidelines for both prevention and treatment of OM, especially in head and neck cancer patients. In pediatric and adult populations, LLLT has been shown to significantly reduce pain intensity and, to a lesser extent, lesion severity [14,18]. Despite its efficacy, the need for specialized equipment and trained personnel restricts its widespread use, particularly in low-resource or outpatient pediatric settings.

Another agent, benzydamine hydrochloride, a topical NSAID rinse, has demonstrated efficacy in the prevention of OM in patients receiving moderate-dose radiotherapy [9]. However, its effectiveness as a treatment for active mucositis is limited, and stinging sensations on inflamed mucosa can reduce patient compliance—especially in children.

Other interventions, including cryotherapy, honey-based formulations, mucosal coating agents, and basic oral care protocols, are variably effective and generally recommended as supportive rather than curative measures.

OROSOL presents a non-pharmacological, topical alternative with a dual mechanical mechanism: (1) an osmotic action that draws hypotonic fluid from the inflamed mucosa to remove surface contaminants (bacteria, necrotic tissue, and proteins) and (2) a mucoadhesive barrier that shields lesions from further irritation and supports healing. Unlike pharmacological agents, OROSOL acts locally, without systemic absorption, minimizing the risk of adverse effects and drug interactions.

In this trial, OROSOL demonstrated rapid and sustained effects, with mucositis scores improving significantly from Day 7 and pain, ulceration, and erythema from Day 14 onward. Feeding difficulty also declined meaningfully from Day 5. These findings are particularly important given the lack of well-tolerated and evidence-based options for managing OM in children.

The broader clinical utility of glycerol-based, filmogenic sprays has been supported in other mucosal conditions, as shown in a recent randomized, placebo-controlled trial of NESOSPRAY HE-C for upper respiratory tract inflammation [19]. In that study, significant symptom relief and excellent tolerability were observed in both children and pregnant women, further supporting the use of this non-systemic, mechanical approach across multiple mucosal sites and vulnerable populations.

Compared to gold-standard therapies such as palifermin, LLLT, and benzydamine, OROSOL offers a practical and accessible solution that does not require systemic delivery, specialized equipment, or medical supervision. It is particularly suited for outpatient pediatric care, where ease of administration and high tolerability are essential. A head-to-head comparison between OROSOL and one of these gold-standard therapies (e.g., LLLT or benzydamine) is planned in future clinical studies to further evaluate its positioning in clinical practice.

This study has several strengths, including its randomized, double-blind design, use of validated endpoints, and consistent efficacy trends across multiple symptoms. However, the sample size was modest, and the follow-up was limited to 28 days. Further research should assess long-term outcomes, recurrence rates, and comparative efficacy in diverse oncologic settings.

## 5. Conclusions

In conclusion, the present study provides robust evidence supporting the use of OROSOL as a safe, effective, and well-tolerated non-pharmacological treatment for oral mucositis in pediatric patients. Its rapid onset of action, sustained symptom relief, and purely mechanical, localized mode of action combined with excellent safety and ease of use make it a strong candidate for integration into routine clinical practice, particularly in settings where systemic treatments are contraindicated or unavailable. OROSOL may serve as a valuable therapeutic option, either as a first-line treatment or in combination with existing supportive care strategies.

## Figures and Tables

**Table 1:** Baseline demographic characteristics of the patient population.

Variable	Value	Group-R	Group-T	*p*-Value
Number of patients	21	10	11	X
Male (M)	M = 7 (33%)	M = 4 (19%)	M = 3 (14%)	0.88
Female (F)	F = 14 (67%)	F = 6 (29%)	F = 8 (38%)	0.88
Age (mean ± SD, [min, max])	9 ± 4 [3,16]	9.9 ± 4.5 [3,16]	8.2 ± 3.6 [3,14]	0.35
Height (mean ± SD, [min, max])	132.7 ± 22.3 [92,171]	137.8 ± 23.8 [95,171]	128.1 ± 21 [92,155]	0.34
Weight (mean ± SD, [min, max])	32.7 ± 14.1 [12.5,63.4]	36.3 ± 16.3 [14.7,63.4]	29.4 ± 11.7 [12.5,48.6]	0.28

## Data Availability

The raw data supporting the conclusions of this article will be made available by the authors on request. The data are not publicly available due to privacy and institutional policy.
